# Biosynthesis of medium-chain length polyhydroxyalkanoates from pre-treated food wastewater

**DOI:** 10.1186/s13068-026-02780-4

**Published:** 2026-06-10

**Authors:** Aghasa Aghasa, Ranaprathap Katakojwala, Eunseok Lee, Sudharshan Juntupally, Hyung-Sool Lee

**Affiliations:** 1 KENTECH Institute for Environmental and Climate Technology, Korea Institute of Energy Technology (KENTECH), 200, Hyeoksin-ro, Naju-si, Jeollanam-do 58330 Republic of Korea; 2 Greeneple Inc., B207-3, Korea Institute of Energy Technology (KENTECH), 21, Kentech-gil, Naju-si, Jeollanam-do 58330 Republic of Korea

**Keywords:** Polyhydroxyalkanoates, Mcl-PHA, Food wastewater, Pseudomonas putida, Ethanol, Sustainable bioplastic

## Abstract

**Background:**

The escalating global plastic waste crisis, coupled with the challenges of managing high-moisture food waste from industrial processing, has prompted nations worldwide to pursue sustainable alternatives and comprehensive waste-reduction strategies. This study addresses this dual challenge by optimizing medium-chain-length polyhydroxyalkanoate (mcl-PHA) production from food wastewater (FWW) using *Pseudomonas putida* through an iterative refinement process, offering a solution that converts waste into biodegradable plastics.

**Results:**

In synthetic substrate tests, the highest PHA yield was 0.70 ± 0.05 g PHA/g VSS, with poly(3-hydroxydecanoate) constituting the majority (77.5 ± 2.6%) of the PHA composition, followed by poly(3-hydroxyoctanoate) (15.6 ± 8.8%). These tests underscored the potential of ethanol as a significant contributor to mcl-PHA biosynthesis. In actual substrate tests using pre-treated soluble FWW (P-FWW), the optimal PHA yield was 0.33 ± 0.02 g PHA/g VSS at an F/M ratio of 5. Lower F/M levels (5–25) predominantly produced short-chain-length PHA (scl-PHA), mainly in the form of poly(3-hydroxybutyrate), while higher F/M levels (50–100) favored mcl-PHA, specifically poly(3-hydroxydecanoate).

**Conclusions:**

This study demonstrates that FWW can be effectively valorized as a sustainable feedstock for PHA production, transforming an industrial waste stream into a valuable biopolymer resource. By establishing the critical relationship between feeding strategy (F/M ratio) and PHA composition, this research provides practical guidelines for optimizing microbial biosynthesis at scale. These findings contribute to circular economy principles by simultaneously addressing food-waste management and reducing dependence on petroleum-based plastics, positioning PHA production from FWW as a viable pathway toward sustainable biomanufacturing.

**Supplementary Information:**

The online version contains supplementary material available at 10.1186/s13068-026-02780-4.

## Background

The escalating global plastic waste crisis has prompted nations to seek sustainable alternatives to conventional plastics. South Korea, in its commitment to environmental stewardship, has set ambitious targets to reduce plastic waste by 20% in 2025 and by 30% in 2030 [1, 2]. Concurrently, the management of food waste, particularly from the food-processing industry which generates approximately 136.412 million cubic meters of wastewater annually as of December 2020, poses a significant challenge [3]. The high-moisture content of food waste, averaging 80%, contributes to wastewater generation as the waste decomposes [4]. Furthermore, the outflow of food-waste solids, ranging from 3 to 13% with foreign materials and 27–33% with wastewater, exacerbates the complexity of waste management [5].

The utilization of food wastewater (FWW) as a feedstock for the biosynthesis of biodegradable plastics, such as polyhydroxyalkanoates (PHA), offers a dual solution to these environmental concerns. PHA is polyesters produced by microbial fermentation, which can be biologically degraded, thus presenting a promising alternative to petrochemical-based plastics [6]. The production of PHA from FWW not only diverts waste from landfills but also contributes to the circular economy by transforming waste into value-added products [7].

*Pseudomonas putida*, a versatile bacterium, has been identified as an efficient producer of PHA [8, 9]. The ability of *P. putida* to metabolize a variety of substrates, including the organic compounds prevalent in FWW, positions it as a suitable candidate for PHA production [10, 11]. However, the presence of active biomass in untreated FWW can interfere with PHA biosynthesis, necessitating a pre-treatment process to enhance substrate solubility and mitigate microbial contamination.

Recent studies have highlighted the effectiveness of thermal pre-treatment with alkali addition in breaking down complex organic compounds into simpler, water-soluble molecules, thereby increasing the availability of substrates for PHA production [12, 13]. This pre-treatment process also deactivates microbial populations, releasing intracellular components that further contribute to solubility [14, 15]. The subsequent separation of soluble products from active biomass is crucial for ensuring a consistent and efficient PHA biosynthesis process.

In this context, the optimization of PHA production from pre-treated soluble FWW (P-FWW) by *P. putida* was investigated, with a focus on the impact of food-to-micro-organism (F/M) ratios and substrate composition. By systematically evaluating the performance of *P. putida* in converting FWW into PHA, it can contribute to the development of sustainable bioplastic production methods that align with South Korea’s waste-reduction goals and the broader global pursuit of sustainable materials management.

## Methods

### Feedstock preparation

- **1.** **Synthetic substrate preparation**

Ethanol, acetic acid, and butyric acid of analytical grade served as representative model compounds for FWW’s predominant components. These chemicals were sourced from Duksan Pure Chemical Co. (Ansan, Republic of Korea).

- **2.** **Pre-treated soluble FWW (P-FWW) preparation**

FWW was obtained from a wastewater treatment plant in Gwangju, Korea. This facility receives mixed food-processing wastewater and food-waste recycling effluents, resulting in an influent stream characterised by fermentation-derived soluble products rather than a single product-specific wastewater. Upon collection, FWW was sieved through a 1-mm mesh to remove larger particulates. The filtered FWW was transferred into a borosilicate glass bottle, filling up to a maximum of 70% of its volume, followed by the addition of NaOH to adjust the pH to 7.5. It was then autoclaved at 121 °C for 15 min. After cooling at 4 °C for at least 6 h, the biomass underwent centrifugation at 10,000 rpm for 10 min. The soluble fraction was separated using vacuum filtration with a 0.45 μm membrane filter, ensuring complete exclusion of the oil fraction and microbial contamination. The resulting soluble feedstock (P-FWW) was stored at 4 °C for future use.

### Detailed experimental design

**Fig. 1:**
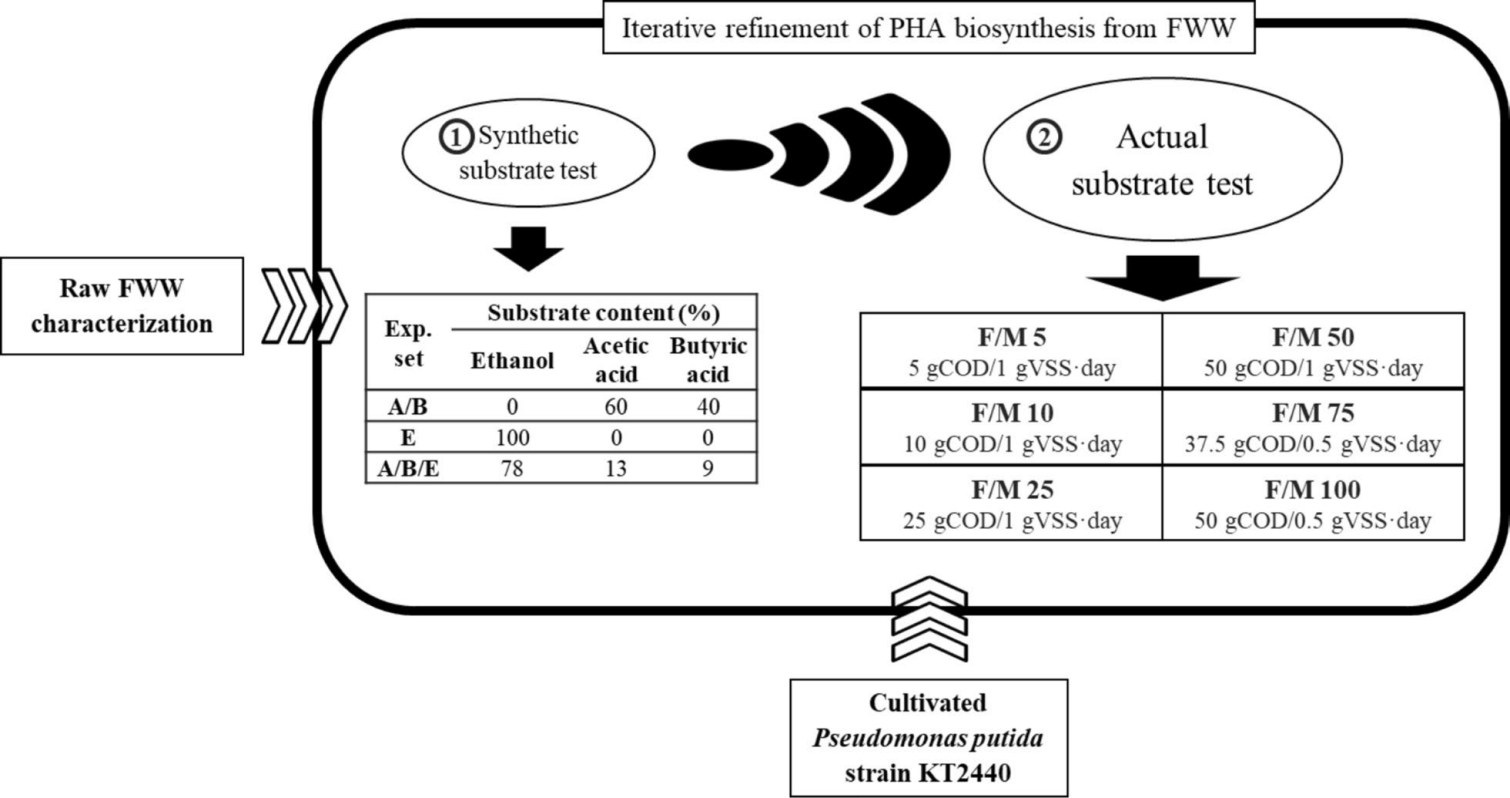
Diagram of the experimental design to evaluate the potential of PHA biosynthesis using FWW as the substrate

PHA biosynthesis in *P. putida* using soluble P-FWW as the substrate underwent iterative refinement across two stages (Fig. 1). In the preliminary stage, *P. putida* was supplied with synthetic substrates mirroring FWW’s composition. To validate which major soluble products in FWW are most effectively utilized for PHA biosynthesis, three synthetic substrate ratios were examined. The A/B/E mixture (78% ethanol, 13% acetic acid, 9% butyric acid on a COD basis) was selected to closely match the measured soluble composition of P-FWW (Table 1). The E condition (100% ethanol) isolated the effect of ethanol alone, whereas the A/B condition (60% acetic acid/40% butyric acid) provided VFAs without ethanol to test PHA production and composition in the absence of alcohol. The food-to-micro-organism (F/M) ratio for this test was consistently set at 5 g COD/g VSS·day across all variations.

**Table 1:** FWW characteristics before and after pre-treatment

Parameters	Unit	Raw FWW	P-FWW	Note
pH	-	3.7 ± 0.3	7.5 ± 0.0	General parameters
COD	g/L	161.9 ± 45.8	109.8 ± 3.7	
sCOD		94.6 ± 9.6	107.1 ± 6.0	
Ethanol	g COD eq./L	26.5 ± 1.5	34.8 ± 0.0	Major product
Acetate		4.1 ± 0.6	5.9 ± 0.5	
Butyrate		0.8 ± 0.1	4.2 ± 0.0	
Propionate	g COD eq./L	0.1 ± 0.0	0.5 ± 0.0	Minor product
Valerate		0.4 ± 0.2	0.8 ± 0.1	

In the final stage, P-FWW was served as the substrate with experiments tailored around varying F/M ratios to gauge FWW’s capability and limit for PHA synthesis. The study assessed six F/M ratios, ranging from 5 to 100 g COD/g VSS·day.

All trials were conducted in an aerobic batch environment using borosilicate glass bottles, each with a 100-mL working volume. Sterilized air was continuously sparged into the bottles using an air pump equipped with 0.2-μm filters. Every condition was triplicated and incubated for 24 h at 30 °C with shaking at 150 rpm.

### Micro-organism and medium

*Pseudomonas putida* strain KT2440 was sourced from Korea Institute of Energy Research (KIER), Gwangju, Republic of Korea. A colony was inoculated in Luria–Bertani (LB) medium (tryptone 10 g/L, yeast extract 5 g/L, NaCl 5 g/L) inside an Erlenmeyer flask. It was incubated at 30 °C overnight with shaking at 150 rpm. Upon a cell population increase, this strain was further cultured for 20 h at the same conditions, using 1% (v/v) of the overnight culture in 1 L of LB medium. The matured cells served as the inoculum for bioplastic synthesis.

### Analytical methods

A gas chromatograph (ACME 6100, YoungIn Chromass, Anyang, Republic of Korea) equipped with an HP-Innowax capillary column (30 m × 0.25 mm × 0.25 μm, Agilent Technologies, USA) and a flame ionization detector (GC–FID) was employed to determine concentrations of ethanol and volatile fatty acids (VFAs), such as acetic, propionic, butyric, valeric, and caproic acids. The oven followed a temperature program: 95 °C for 2 min, ramping to 140 °C at 10°C/min for another 2 min, and then escalating to 240 °C at 40°C/min for 5 min. The injection conditions were 240 °C with a split mode at a 10:1 ratio and a flow rate of 1.0 mL/min, utilizing helium as the carrier gas.

Soluble chemical oxygen demand (sCOD) and biomass/volatile suspended solids (VSS) concentrations were measured with Standard Methods [16]. Samples underwent filtration through Sartorius Minisart^®^ 0.2 μm (Göttingen, Germany) for soluble measurements, including VFAs and sCOD evaluations. All measurements were conducted in triplicate.

### PHA quantification and analysis

The following approach, modified from the previous study [17], was employed for biomass isolation and PHA analysis. Biomass activity was halted using 1% (v/v) formaldehyde for precise PHA estimation in *P. putida* KT2440 cultivated under varied conditions. After centrifugation at 10,000 rpm for 10 min, supernatants were discarded, and the resultant biomass pellets underwent distilled water rinsing, followed by further centrifugation. These washed pellets were oven-dried at 85 °C for a day. A 15 mg sample of the pellets was introduced to a blend of 2 mL chloroform and 2 mL of acidified methanol (1:1 ratio, with 3% v/v H_2_SO_4_ in the methanol) containing 100 mg/L sodium benzoate as an internal standard.

Four reference solutions were prepared as follows: 50 mg of Poly(3-hydroxybutyric acid–co-3-hydroxyvaleric acid) for detecting scl-PHA, represented by poly(3-hydroxybutyrate) (PHB) and poly(3-hydroxyvalerate) (PHV); 20 μL methyl 3-hydroxyhexanoate; 5 mg (±)−3-hydroxyoctanoic acid; and 6 mg (±)−3-hydroxydecanoic acid to identify mcl-PHA, specifically Poly(3-hydroxyhexanoate) (PHHx), poly(3-hydroxyoctanoate) (P3HO), and poly(3-hydroxydecanoate) (P3HD). Each solution was dissolved in 15 mL of chloroform. All standards were obtained from Millipore Sigma (Sigma-Aldrich). Samples and standards were digested in sealed 10 mL glass vials at 100 °C for 3.5 h and then cooled to room temperature. The process continued with the mixing of samples with distilled water, followed by centrifugation, and filtration through a 0.2 μm filter to isolate the chloroform phase [18, 19].

For the methanolized PHA sample analysis, GC–FID was utilized at an operational temperature of 300 °C and an injection port setting of 250°C. With helium as the carrier gas, a 1:15 split injection ratio was maintained. The oven was started at 50 °C for 5 min, then was increased to 250 °C at 20°C/min, holding for an additional 10 min. PHA monomer quantification relied on a standard curve. Monomer concentrations were quantified against a five-point external calibration curve prepared from the reference standards described above (R^2^ > 0.99 for all monomers). The identity of each peak was confirmed by comparison of retention times with authenticated standards [17, 20].

PHA yield was calculated as the ratio of the total intracellular PHA mass (g) quantified by GC–FID to the total biomass volatile suspended solids (VSS, g) measured at the end of the 24-h batch test, expressed as g PHA/g VSS. VSS, measured according to Standard Methods, represents total biomass, including PHA granules [17, 21].

### PHA pellet extraction and characterization

A method modified from previous studies was utilized for PHA pellet extraction [22, 23]. Biomass underwent centrifugation at 10,000 rpm for 10 min. The resulting pellet was cleaned with Phosphate Buffer Saline (PBS) via centrifugation at 10,000 rpm for 10 min to eliminate surface contaminants. The biomass was then treated with an equal volume of 10% sodium hypochlorite and incubated at 37 °C with shaking at 120 rpm for 1.5 h. Following this, the biomass was rinsed with PBS, mixed with an equal volume of chloroform, and subjected to digestion in a sealed vessel using a water bath at 70 °C for 15 min. The chloroform layer was extracted and introduced to ice-cold methanol to precipitate the PHA, which was subsequently centrifuged and dried for further analyses. Functional group characterization of the extracted PHA was conducted at room temperature via Fourier transform infrared (FTIR) spectroscopy using a Nicolett 6700 from Thermo Scientific.

### Statistical analysis

All experimental data are presented as mean values ± standard deviation (SD) from triplicate measurements (n = 3). Correlation and regression analysis were performed to quantify relationships between experimental conditions (substrate composition in the synthetic substrate test and F/M ratio in the actual substrate test) and PHA production parameters. Pearson correlation coefficients (*r*) were calculated to assess the strength and direction of bivariate relationships, with statistical significance assessed at *α* = 0.05 (two-tailed). Linear regression analysis using least-squares fitting was employed to establish predictive equations for key parameters as functions of the independent variables. The coefficient of determination (*R*^*2*^) was calculated to evaluate the proportion of variance explained by each regression model. Statistical analyses were conducted using Python 3.14.2 with scipy.stats library (version 1.16.2). The complete analysis report and codebase are available in the Supplementary Material.

## Results

### Pre-treatment of FWW

Before utilizing FWW as a substrate for PHA production, its composition was analyzed as a preliminary step. As shown in Table 1, ethanol was the predominant soluble component in the raw FWW (26.5 ± 1.5 g COD eq./L), followed by acetate (4.1 ± 0.6 g COD eq./L) and butyrate (0.8 ± 0.1 g COD eq./L), with propionate and valerate present only in minor amounts. After the pre-treatment, these soluble products increased further in P-FWW (ethanol 34.8 ± 0.0, acetate 5.9 ± 0.5, butyrate 4.2 ± 0.0 g COD eq./L), confirming that fermentation-derived alcohols and VFAs are the main carbon sources feeding PHA biosynthesis.

It should be noted that the composition of FWW can vary seasonally and across collection sites. The values reported in Table 1 represent the specific batch used in this study, and compositional variability in real-world FWW streams may influence PHA yields and compositions.

Post–pre-treatment, the P-FWW was deemed suitable for direct PHA biosynthesis in actual substrate tests. However, the viability and efficacy of the feedstock warrant validation via synthetic substrate tests in the subsequent phase.

### Synthetic substrate test results

**Fig. 2:**
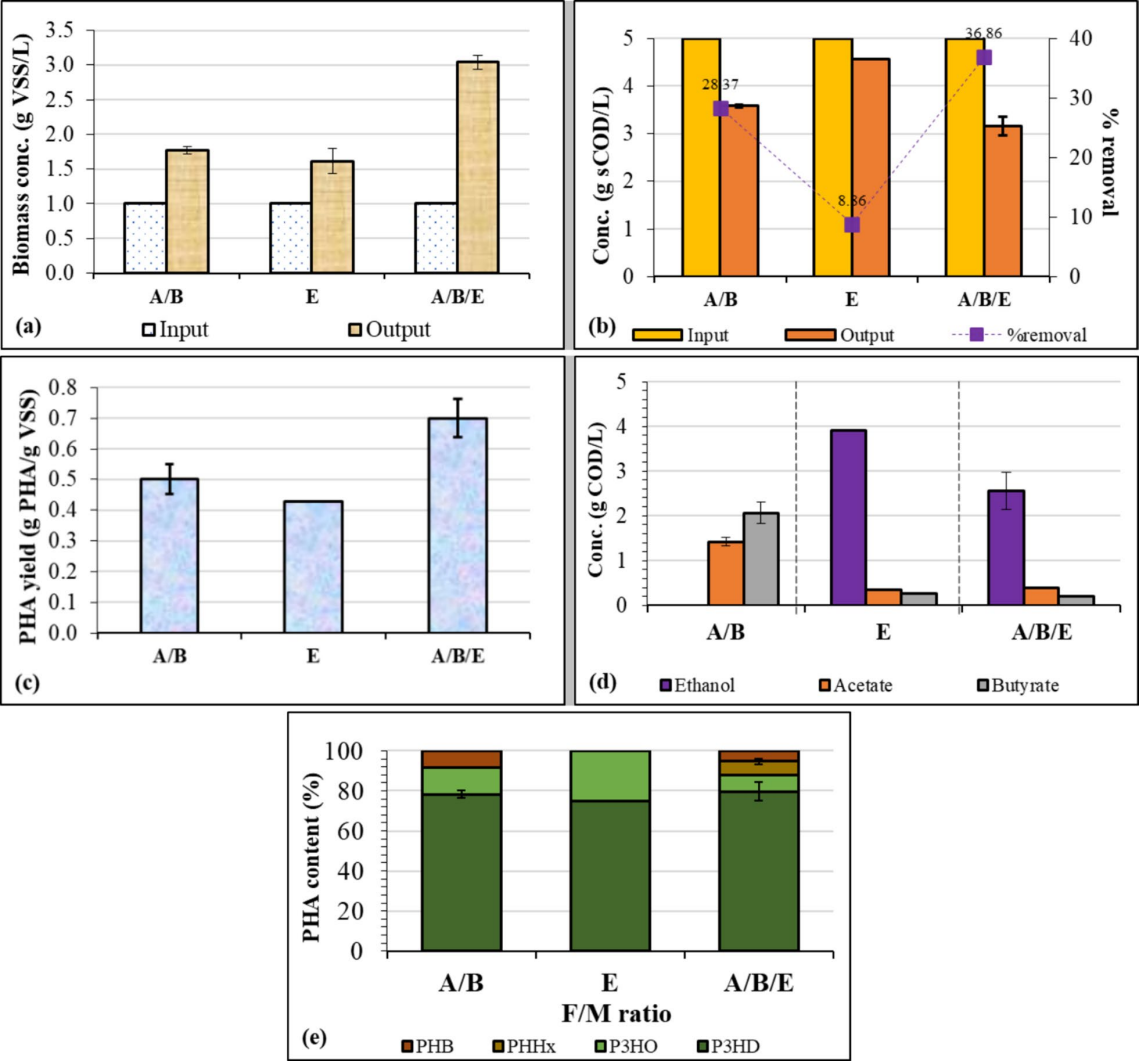
Synthetic substrate test analysis results on **(a)** biomass concentration, **(b)** sCOD, **(c)** PHA yield, **(d)** residual ethanol + VFAs, and (**e**) PHA content. All data are means ± SD (n = 3)

In this study, synthetic substrates were utilized to evaluate FWW’s potential as the primary substrate for PHA biosynthesis, emphasizing degradation efficiency and the resultant PHA yield and content. As illustrated in Fig. 2a, biomass concentration peaked at 3.1 ± 0.1 g VSS/L, marking a threefold augmentation from the initial concentration. The maximum sCOD degradation rate reached 36.9 ± 3.8% (Fig. 2b), with the highest yield recorded at 0.70 ± 0.05 g PHA/g VSS (Fig. 2c); this result is close to or higher than the maximum PHA yield from FWW to our knowledge [17, 24, 25].

Based on the residual Ethanol + VFAs results (Fig. 2d), set E and A/B/E were predominantly generated acetate and butyrate from ethanol degradation (Fig. 2d).

On the other hand, in analyzing PHA content (Fig. 2e), mcl-PHA was mostly observed across all sets. Specifically, P3HD recorded the highest percentage at 77.5 ± 2.6%, trailed by P3HO, which averaged 15.6 ± 8.8% across the three synthetic substrate sets.

### Actual substrate test results

**Fig. 3:**
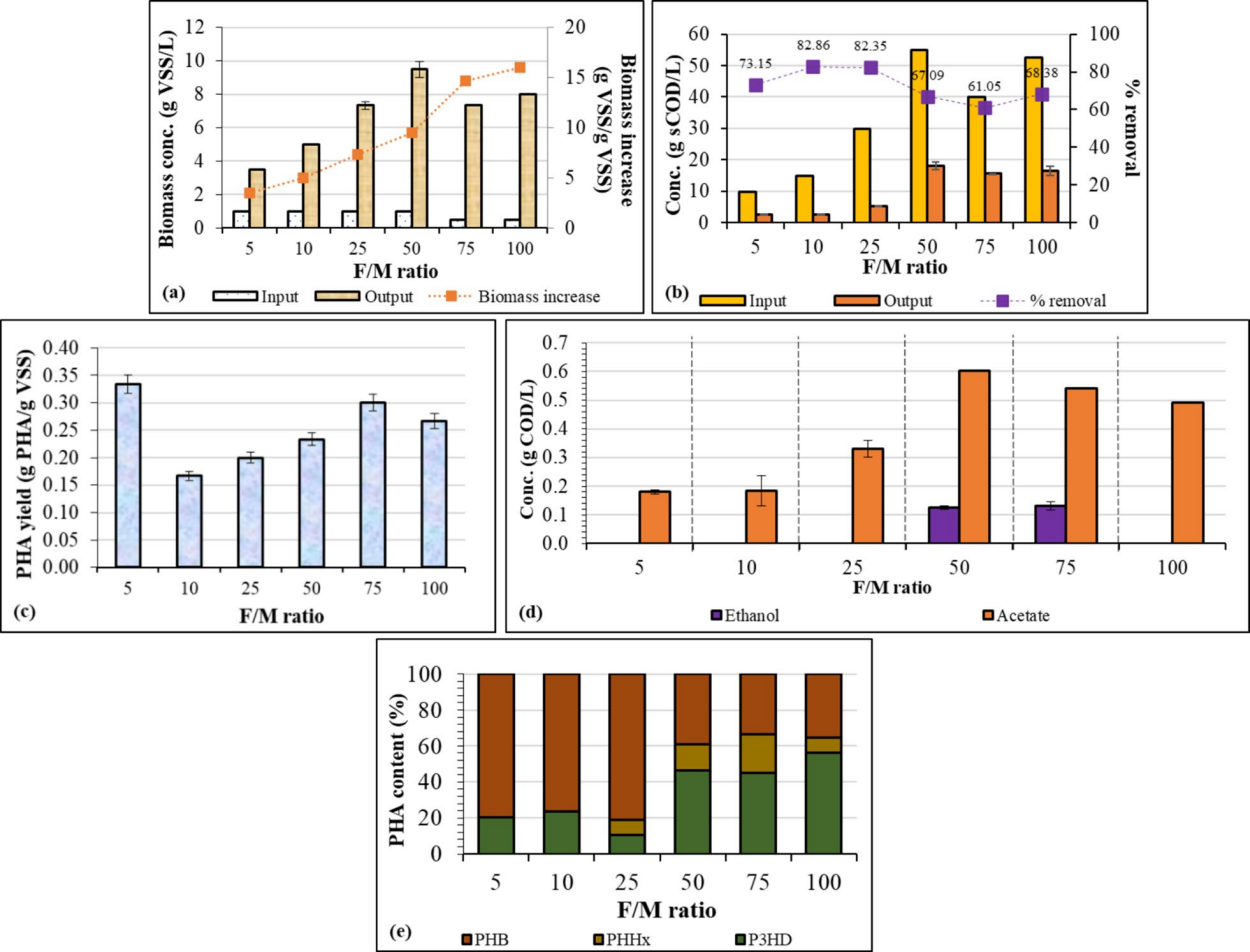
Actual substrate test analysis results on **(a)** biomass concentration, **(b)** sCOD, **(c)** PHA yield, **(d)** residual ethanol + VFAs, and (**e**) PHA content. All data are means ± SD (n = 3)

Utilizing six distinct F/M ratios illuminated the potential of FWW as an alternative substrate for PHA production, consistent with earlier synthetic substrate tests. Figure 3 elucidates varying outcomes across parameters. Specifically, F/M 100 registered the highest biomass concentration increase—a 15-fold enhancement (Fig. 3a). Conversely, the peak sCOD degradation rate was achieved by F/M 10 at 82.9 ± 0.1% (Fig. 3b), and F/M 5 yielded the highest PHA at 0.33 ± 0.02 g PHA/g VSS (Fig. 3c).

Regarding the residual ethanol + VFAs profile (Fig. 3d), ethanol was fully metabolized on almost all F/M ratio gradations, yet acetate persisted post the 24-h reaction in all cycles.

In terms of PHA composition (Fig. 3e), a unique profile emerged. Lower F/M levels (F/M 5–25) predominantly yielded scl-PHA in the form of PHB, while elevated levels (F/M 50–100) favored mcl-PHA, specifically P3HD.

To gauge the commercial viability of the resultant PHA, comprehensive characterization is imperative in subsequent stages.

### FTIR analysis of PHA functional groups

**Fig. 4:**
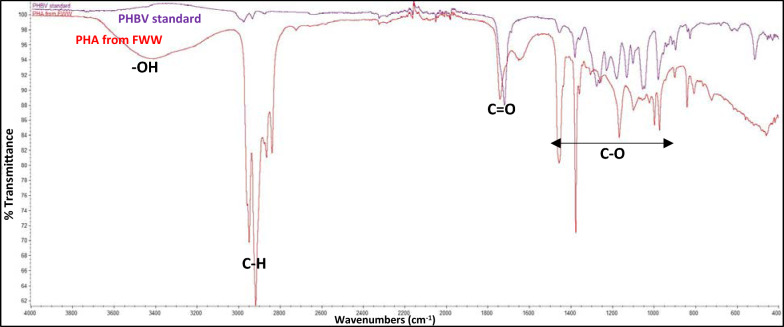
FTIR spectrum of PHA produced from FWW biosynthesis compared to the PHBV standard

To discern the functional groups present in the PHA, FTIR analysis was conducted (Fig. 4) on the F/M 100 sample specifically as an example in this study. The resultant spectra of the extracted PHA displayed characteristic absorption bands: C–H stretching of the methylene group around 2900 cm⁻^1^, C–H bending vibrations at 1454 cm⁻^1^ and 1379 cm⁻^1^, and a dominant absorption peak at 1724 cm⁻^1^, which is characteristic of the ester group’s carbonyl carbon (C = O bond). In addition, the C–O stretching of the ester group was represented by the band spanning 970–1275 cm⁻^1^, while bands spanning 1450–900 cm⁻^1^ were attributed to ether linkage stretching. The PHA sample derived from P-FWW exhibited all characteristic bands signifying C–H and carbonyl stretching, consistent with the PHA biopolymer structure.

The PHA sample derived from FWW exhibited notable differences in spectral characteristics compared to that of the PHBV standard. A perceptible shift in transmittance was observed at approximately 1725 cm⁻^1^ (C = O group), with a slight movement toward higher wavenumber regions. Absorption bands within the 1500–800 cm⁻^1^ range showed subtle transitions toward lower wavenumbers. Bands consistent with crystalline (approximately 1300 cm⁻^1^) and amorphous domains (approximately 1150 cm⁻^1^) were evident. These spectral attributes confirmed the presence of polyhydroxy groups characteristic of the PHA biopolymer, with distinct spectroscopic signatures between the samples derived from different substrate types.

### Statistical analysis results

**Fig. 5:**
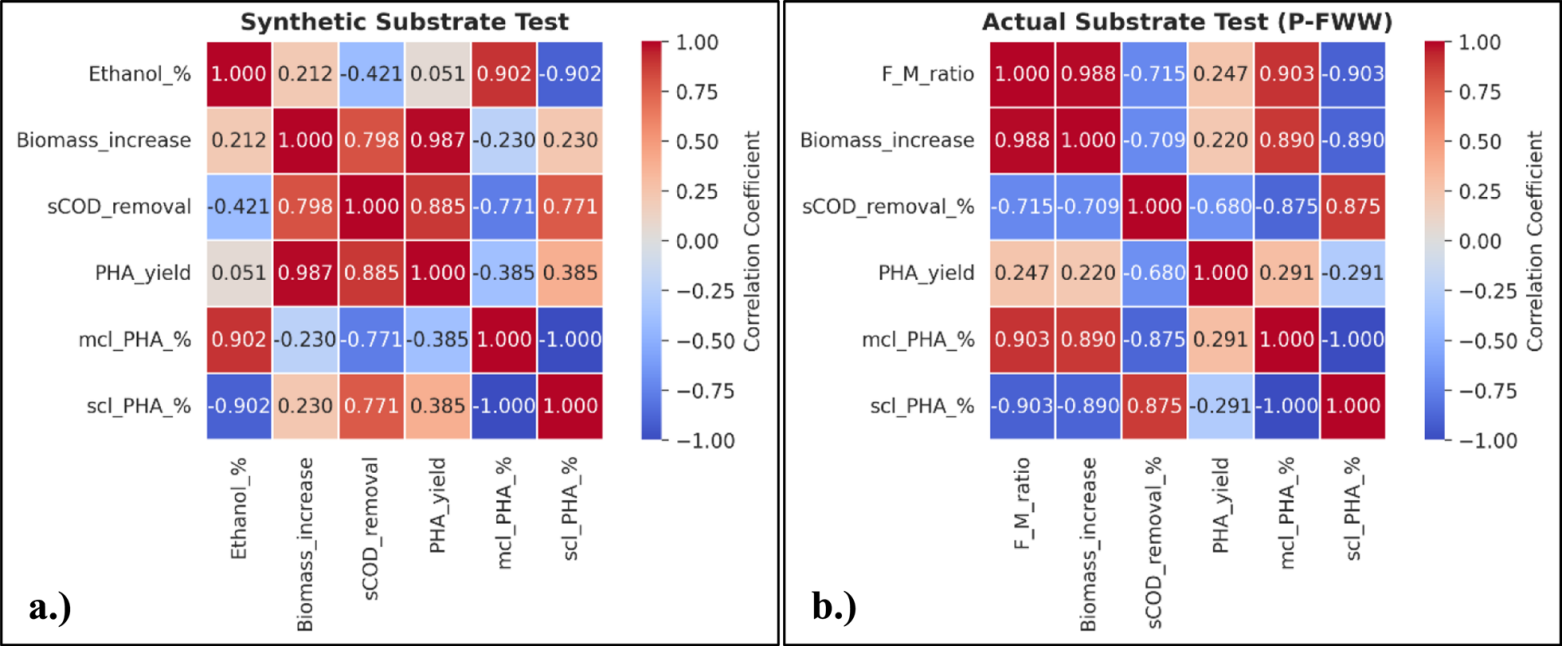
Correlation analysis matrices heatmap of (**a)** synthetic substrate test and (**b)** actual substrate test

Correlation analysis revealed distinct relationships between experimental parameters and performance metrics in the two substrate tests (Fig. 5). In the synthetic substrate test (Fig. 5a), ethanol content showed a strong positive correlation with mcl-PHA composition (*r* = 0.9023) and a strong negative correlation with scl-PHA composition (*r* = −0.9023). However, linear regression analysis revealed no statistically significant relationships with any performance metric (*p* > 0.05) (Fig. S1).

In the actual substrate test, the correlation analysis of the actual substrate test revealed several key relationships between F/M ratio and measured variables (Fig. 5b). The F/M ratio showed a very strong positive correlation with biomass increase (*r* = 0.9876), indicating that feeding strategy directly influences bacterial growth. Conversely, the F/M ratio showed a negative correlation with sCOD removal percentage (*r* = −0.7147), suggesting that higher feeding rates reduce the relative extent of substrate degradation. In addition, the F/M ratio demonstrated a strong positive correlation with mcl-PHA composition (*r* = 0.9030) and a correspondingly strong negative correlation with scl-PHA composition (*r* = −0.9030), indicating that polymer chain length is tightly coupled to substrate availability.

Regarding the linear regression analysis in the actual substrate test (Fig. S2), F/M ratio also demonstrated strong predictive relationships with multiple performance parameters. Most notably, F/M ratio exhibited an extremely strong relationship with biomass increase (*R*^*2*^ = 0.9753, *p* = 0.0002). In addition, mcl-PHA content increased significantly with F/M ratio (*R*^*2*^ = 0.8155, *p* = 0.0136), while scl-PHA content showed the inverse relationship (*R*^*2*^ = 0.8155, *p* = 0.0136). In contrast, F/M ratio did not significantly predict PHA yield and sCOD removal (*p* > 0.05), suggesting that other operational factor control might control the values.

## Discussion

### Impact of pre-treatment processes on FWW characteristics

Typically, FWW samples from Korea predominantly contain fermented food products with varying consistencies from liquid to semi-solid [26, 27]. According to Table 1, such a fermentation profile, with ethanol as a dominant product, generally emerges at pH levels below 5 [28–30]. This observed characteristic of the FWW is particularly beneficial, since ethanol is a viable substrate for mcl-PHA synthesis by *P. putida* [21, 31]. However, due to the fermentation of the feedstock, active biomass remains present, which, if untreated, could impact PHA biosynthesis. Therefore, a separation of the soluble products and active biomass was deemed necessary.

In terms of pre-treatment, while some studies vouch for the efficacy of autoclaving in sterilizing biomass under high-pressure steam conditions [32], the increased solubility of P-FWW might also be attributed to the prior addition of an alkali substance (i.e., NaOH). It has been previously reported that the integration of an alkali buffer during thermal pre-treatment accelerates the breakdown of complex organic compounds (such as proteins, carbohydrates, and lipids) into simpler, water-soluble molecules [33, 34]. Concurrently, this process deactivates the microbial populations in FWW by disrupting cellular structures, which, in turn, release intracellular components, further augmenting solubility [12]. Furthermore, the borosilicate containment during pre-treatment effectively preserved the volatile components post-cooling. Regarding the tCOD, its concentration diminished due to vacuum filtration, which effectively separated the oil and residual biomass after FWW’s pre-treatment.

It should be noted that the present work focuses on the soluble pre-treated fraction (P-FWW); direct comparison with raw FWW, which still contains active biomass and suspended solids, remains an important subject for future work and will be required to fully quantify the trade-off between pre-treatment intensity, PHA yield, and process robustness.

### Evaluation of PHA biosynthesis via synthetic substrate test

Notably, the A/B/E set outperformed the A/B and E sets in metrics of biomass concentration, sCOD degradation, and PHA yield (Fig. 2a–c). This aligns with prior findings, suggesting that the integration of different soluble products, predominantly ethanol, can enhance PHA biosynthesis in *P. putida* [35, 36]. Correlation analysis of the synthetic substrate test (Fig. 5a) revealed a strong positive relationship between ethanol content and mcl-PHA composition (*r* = 0.9023), indicating that ethanol-rich substrates inherently promote longer chain synthesis. Although regression analysis did not yield statistically significant relationships (*p* > 0.05), the strong correlation coefficient and the improved performance of the A/B/E set provide preliminary evidence supporting substrate composition's role in PHA type selection.

Regarding the phenomenon in ethanol + VFAs profile (Fig. 2d), this could happen due to ethanol metabolism into acetaldehyde, releasing fatty acids during the transformation, which can subsequently partake in the PHA biosynthesis pathway [31]. The correlation analysis further supports this mechanism, showing that biomass increase and sCOD removal were strongly correlated (*r* = 0.7975) in the synthetic tests, suggesting coordinated substrate utilization for both growth and polymer synthesis.

The analysis results related to the PHA content (Fig. 2e) suggest that *P. putida* might preferentially produce mcl-PHA from ethanol and VFAs due to its more direct synthesis pathway, wherein (R)−3-hydroxyacyl-CoA from the β-oxidation process can immediately interact with mcl-PHA synthase, facilitating mcl-PHA production [37]. The role of ethanol in promoting mcl-PHA formation was assessed through a direct controlled comparison of three substrate sets at identical F/M conditions (5 g COD/g VSS·day). Sets containing ethanol (E and A/B/E) both yielded higher mcl-PHA fractions than the ethanol-free A/B set (Fig. 2e), and correlation analysis confirmed a strong positive relationship between ethanol content and mcl-PHA composition (r = 0.9023). Although regression analysis did not reach statistical significance due to the small sample size (n = 3), these controlled comparisons provide experimental evidence—not merely compositional inference—that ethanol availability promotes mcl-PHA synthesis, consistent with its established entry into the β-oxidation pathway via acetaldehyde [7, 31].

The outcomes solidify the notion that a combination of ethanol, acetate, and butyrate as primary soluble products is advantageous for PHA biosynthesis. These findings also underscore the potential of FWW as a sustainable substrate for mcl-PHA synthesis by *P. putida*. Thus, venturing into actual substrate testing will further elucidate FWW’s potential in mcl-PHA biosynthesis.

### Optimization of PHA biosynthesis from FWW

An emerging pattern suggests that elevated F/M ratios drive biomass growth (Fig. 3a). Regression analysis confirmed this observation with an exceptionally strong relationship between F/M ratio and biomass increase (*R*^*2*^ = 0.9753, *p* = 0.0002). This relationship explained 97.5% of the observed variation, indicating that F/M ratio is the dominant predictor of bacterial growth. In addition, lower F/M ratios correlate with heightened sCOD removal (Fig. 3b), as confirmed by a negative correlation (*r* = − 0.7147) between F/M ratio and sCOD removal percentage. This trend may be attributed to the nutrient abundance in P-FWW; the inherent nutrient richness in FWW is conducive for microbial growth [38, 39]. Therefore, administering increased volumes of P-FWW could be identical to introducing surplus nutrients, thereby promoting biomass proliferation [40].

As for the ethanol + VFAs profile (Fig. 3c), the observation aligns with prior synthetic substrate test outcomes, reinforcing the notion that *P. putida* may favor ethanol over acetate as a substrate [41]. Correlation analysis further supports this finding, showing a very strong positive correlation between F/M ratio and mcl-PHA composition (*r* = 0.9030), indicating that substrate availability directly influences polymer chain length selection in a manner consistent with the synthetic substrate test results.

Interestingly, maximum PHA yield was observed at the lowest F/M ratio (Fig. 3d), even though both statistical analyses revealed that the F/M ratio did not significantly predict PHA yield, suggesting that factors other than substrate quantity control absolute yield. However, certain studies indicate that F/M ratios can influence cellular redox balance [42]. At reduced F/M levels, cells might experience stress due to carbon scarcity. A pivotal factor in amplifying PHA biosynthesis hinges on the micro-organisms’ adeptness to exploit excess cellular electrons, a phenomenon observable under stress conditions [43, 44]. Thus, the carbon deficit in the F/M 5 system could potentially catalyze PHA production.

Regarding the varied PHA composition from different levels of F/M ratio, although the conditions to stimulate PHA biosynthesis are somewhat uniform, microbial responses can vary. Regression analysis demonstrated a significant relationship between F/M ratio and PHA composition (*R*^*2*^ = 0.8155, *p* = 0.0136). This bidirectional compositional control indicates a clear metabolic threshold around F/M = 25–50, where the system shifts from scl-PHA dominance to mcl-PHA dominance. Literature suggests *P. putida* may produce scl-PHA under carbon-deficient conditions to mitigate redox stress [42], a mechanism supported by the strong negative correlation between F/M ratio and scl-PHA content (*r* = − 0.9030). The shift from scl-PHA to mcl-PHA at higher F/M ratios can be understood through the metabolic pathways of *P. putida*. Under carbon-limiting conditions (low F/M), the primary substrate flux is directed through acetyl-CoA to the PHB biosynthesis pathway, favoring shorter-chain monomers [8, 45]. At higher F/M ratios, increased substrate availability activates the β-oxidation pathway more intensively, generating (R)−3-hydroxyacyl-CoA intermediates of longer chain lengths that are directly channeled to mcl-PHA synthase [8]. This metabolic switching mechanism explains the clear compositional threshold observed around F/M = 25–50 in the results.

A notable difference emerged between the synthetic substrate tests and the actual P-FWW tests. In the synthetic tests, the maximum PHA yield reached 0.70 ± 0.05 g PHA/g VSS under an F/M ratio of 5 g COD/g VSS·day (Fig. 2c), whereas the highest yield obtained with P-FWW was 0.33 ± 0.02 g PHA/g VSS (Fig. 3c). This discrepancy can be attributed to several factors. First, the synthetic substrates contained only readily assimilable ethanol and VFAs at controlled ratios, whereas P-FWW also includes nonfermentable or slowly degradable organic matter and other components that do not contribute directly to PHA storage but still consume oxygen and nutrients. Second, only a fraction of the COD in P-FWW is present as ethanol and VFAs (Table 1), so increasing the F/M ratio primarily increases total COD rather than the fraction of readily storable carbon. Third, our regression analysis indicates that F/M ratio strongly predicts biomass accumulation and PHA monomer composition (*R*^*2*^ = 0.9753 for biomass increase and 0.8155 for mcl-PHA content), but not PHA yield (*R*^*2*^ = 0.061), suggesting that additional operational factors beyond substrate quantity limit the conversion of biomass into polymer. Together, these effects could explain why the defined synthetic substrates produce higher yields under idealized conditions, while P-FWW offers lower yield but more realistic process behavior.

### FTIR evidence of polymer composition and structure

FTIR spectral analysis provided detailed molecular-level insights into the PHA structures produced from both substrate types. The characteristic carbonyl absorption at 1724 cm⁻^1^ is consistent with values reported in prior studies on PHA [46, 47], confirming the presence of functional polyester groups, regardless of the substrate source. The presence of the full complement of absorption bands (C–H stretching, C–O stretching, and ether linkages in both crystalline and amorphous domains) substantiates the structural integrity of the produced biopolymer.

The observable spectral shift in the carbonyl region (around 1725 cm⁻^1^) for the FWW-derived sample, coupled with transitions in the lower wavenumber regions (1500–800 cm⁻^1^), suggests compositional differences between the samples. These alterations could be influenced by intermolecular hydrogen bonding between the carbonyl groups and hydroxyl entities within the polymer structure, a phenomenon more pronounced in mcl-PHA due to the presence of longer side chains. This spectroscopic evidence aligns with the regression analysis findings (*R*^*2*^ = 0.8155, *p* = 0.0136), demonstrating that the F/M ratio controls polymer chain length selection. The convergence of spectroscopic data with statistical findings validates that the substrate feeding strategy directly influences not only the biosynthetic pathway but also the molecular structure and intermolecular interactions of the resulting polymer.

It is acknowledged that FTIR alone cannot unambiguously determine monomer composition or polymer sequence distribution; for that purpose, nuclear magnetic resonance (NMR) spectroscopy would provide more definitive structural characterization [48]. The monomer identity and relative proportions reported in this study were established primarily by GC–FID using authenticated reference standards for each individual monomer (see Methods section), with FTIR serving as a complementary confirmation of functional group chemistry. Future studies should incorporate NMR to provide complete polymer structural characterization.

### Implications for sustainable biopolymer production and industrial application

This study has several important implications for PHA production as a sustainable biopolymer alternative. The successful production of mcl-PHA from food wastewater addresses a critical challenge in the circular bioeconomy: converting low-value organic waste into high-value biochemical products. The quantitative control of composition through F/M ratio provides a practical operational parameter for bioprocess optimization, enabling producers to design PHA compositions tailored to specific application requirements. With regression equations established for biomass accumulation and polymer composition at laboratory-scale, predictive modeling of reactor behavior represents a useful starting point for future process design, although validation under pilot- or industrial-scale conditions will be necessary.

Furthermore, the identification of a clear metabolic threshold at F/M = 25–50 represents a critical control point for the selective manufacture of scl-PHA or mcl-PHA. This compositional control is significant, because different PHA chain lengths possess distinct material properties: scl-PHA typically exhibits higher crystallinity, melting temperature, and mechanical strength, making it suitable for rigid applications, whereas mcl-PHA demonstrates greater elasticity and flexibility, expanding potential applications in elastomeric products [21, 49]. The ability to modulate these properties through a single operational parameter (F/M ratio) without requiring substrate modification enhances the economic viability of FWW-based PHA production processes.

However, since P-FWW investigated in this study originates from a treatment plant receiving mixed food-processing and food-waste recycling effluents, the obtained results represent an aggregate behavior rather than industry-specific responses. Future studies should extend this approach to FWW from well-defined processing sectors (e.g., dairy, beverage, or fermented food production) to validate and refine the operational guidelines derived from this work.

Collectively, this study strengthens the broader narrative in the field that food-waste substrates, despite compositional variability, can be reliably processed for biochemical production when coupled with appropriate microbial physiology and process control. The convergence of observational findings, statistical relationships, and spectroscopic evidence (i.e., FTIR) validates that *P. putida* does not merely tolerate FWW as a substrate but actively exploits its compositional characteristics for enhanced polymer production. This shifts the paradigm from FWW as a waste disposal challenge to FWW as a valuable feedstock for high-performance biopolymer synthesis. As global interest in bio-based polymers intensifies due to environmental concerns and regulatory pressures on petrochemical plastics, these findings provide essential evidence that sustainable, waste-derived PHA production is technically feasible and operationally controllable at scales approaching industrial relevance.

## Conclusions

The potential of utilizing FWW as a substrate for PHA production with *P. putida* was successfully demonstrated. The synthetic substrate tests yielded a maximum PHA production of 0.70 ± 0.05 g PHA/g VSS, dominated by mcl-PHA. In actual substrate tests, the optimal PHA yield was 0.33 ± 0.02 g PHA/g VSS at an F/M ratio of 5. The findings also indicate that lower F/M ratios primarily produce scl-PHA, whereas higher F/M ratios favor mcl-PHA. These results, obtained at laboratory scale, demonstrate the technical feasibility of FWW as a substrate for PHA biosynthesis and highlight the importance of substrate composition and feeding strategy in controlling PHA yield and composition. Future work should validate these findings at larger scales and under continuous operating conditions.

## Supplementary Information

Supplementary Material 1.

## Data Availability

The datasets generated and analyzed during the current study, including raw measurements of VSS, sCOD, PHA monomer concentrations, and the complete Python analysis codebase, are available from the corresponding author upon reasonable request.
