# Supplementary material for: Biosynthesis of medium-chain length polyhydroxyalkanoates from pre-treated food wastewater
Source: Biotechnol Biofuels Bioprod. 2026 Jun 10;19:62. doi: 10.1186/s13068-026-02780-4 (PMC13474916; doi:10.1186/s13068-026-02780-4)
Supplement: Supplementary file 1 [file 13068_2026_2780_MOESM1_ESM.docx]

Biosynthesis of medium-chain length polyhydroxyalkanoates from pre-treated food wastewater

Aghasa Aghasa^1,2^, Ranaprathap Katakojwala^2^, Eunseok Lee^1^, Sudharshan Juntupally^2^, and Hyung-Sool Lee^1,2,§^

^1^KENTECH Institute for Environmental and Climate Technology, Korea Institute of Energy Technology (KENTECH), 200, Hyeoksin-ro, Naju-si, Jeollanam-do, Republic of Korea

^2^Greeneple Inc., C305, Korea Institute of Energy Technology (KENTECH), 21, Kentech-gil, Naju-si, Jeollanam-do, 58330, Republic of Korea

^§^Corresponding author. E-mail: [hs.chris.lee@kentech.ac.kr](mailto:hs.chris.lee@kentech.ac.kr)

**Summary of supplementary materials**

S1. Python codebase details and analysis reports on correlation and regression analysis

S1.1 Details on phyton codebase

S1.2 Analysis reports and generated materials

# S1. Python codebase details and analysis reports on correlation and regression analysis

## S1.1 Details on python codebase

The following python codebase was employed using the data sets of the synthetic substrate test and the actual substrate test. For the synthetic substrate test, ethanol content was analyzed as the independent variable. For the actual substrate test, food-to-microorganism (F/M) ratio was analyzed as the independent variable. Response variables included PHA yield, biomass concentration, sCOD removal rate, and PHA monomeric composition. The code was written as follows:

import pandas as pd

import numpy as np

import matplotlib.pyplot as plt

import seaborn as sns

from scipy.stats import linregress, pearsonr

import warnings

warnings.filterwarnings('ignore')

\# ============================================================================

\# STEP 1: DATA PREPARATION

\# ============================================================================

\# Synthetic Substrate Test Data

synthetic_data = {

'Set': ['A/B', 'A/B/E', 'E'],

'Acetate_%': [60, 9, 0],

'Butyrate_%': [40, 13, 0],

'Ethanol_%': [0, 78, 100],

'Biomass_0h': [1, 1, 1],

'Biomass_24h': [1.770308, 3.039916, 1.615546],

'sCOD_0h': [5, 5, 5],

'sCOD_24h': [3.581683, 3.15681, 4.557042],

'PHA_yield': [0.501127, 0.7, 0.428339],

'scl_PHA_%': [8.434892, 5.195326, 0],

'mcl_PHA_%': [91.565108, 94.804674, 100.0]

}

synthetic_df = pd.DataFrame(synthetic_data)

\# Calculate additional variables for synthetic substrate test

synthetic_df['Biomass_increase'] = synthetic_df['Biomass_24h'] - synthetic_df['Biomass_0h']

synthetic_df['sCOD_consumed'] = synthetic_df['sCOD_0h'] - synthetic_df['sCOD_24h']

synthetic_df['sCOD_%_removal'] = (synthetic_df['sCOD_consumed'] / synthetic_df['sCOD_0h']) * 100

\# Actual Substrate Test Data

actual_data = {

'F_M_ratio': [5, 10, 25, 50, 75, 100],

'Biomass_0h': [1, 1, 1, 1, 0.5, 0.5],

'Biomass_24h': [3.5, 5, 7.333333, 9.5, 7.333333, 8.0],

'VSS_increase': [3.5, 5, 7.333333, 9.5, 14.666667, 16.0],

'sCOD_0h': [9.893249, 14.893249, 29.893249, 54.893249, 39.946624, 52.446624],

'sCOD_24h': [2.656012, 2.553427, 5.275203, 18.06266, 15.558372, 16.58484],

'COD_%_removal': [73.153283, 82.855138, 82.353198, 67.094933, 61.052097, 68.377679],

'PHA_yield': [0.333333, 0.166667, 0.2, 0.233333, 0.3, 0.266667],

'scl_PHA_%': [79.902855, 76.489476, 81.152503, 38.991503, 33.45828, 35.152332],

'mcl_PHA_%': [20.097145, 23.510524, 18.847497, 61.008497, 66.54172, 64.847668]

}

actual_df = pd.DataFrame(actual_data)

print("Data loaded successfully!")

print(f"Synthetic test shape: {synthetic_df.shape}")

print(f"Actual test shape: {actual_df.shape}")

\# ============================================================================

\# STEP 2: SYNTHETIC SUBSTRATE TEST - CORRELATION ANALYSIS

\# ============================================================================

print("\n" + "="*100)

print("SYNTHETIC SUBSTRATE TEST - CORRELATION AND REGRESSION ANALYSIS")

print("="*100)

\# Variables for analysis in synthetic test

synthetic_analysis = {

'Ethanol_%': synthetic_df['Ethanol_%'].values,

'Biomass_increase': synthetic_df['Biomass_increase'].values,

'sCOD_removal': synthetic_df['sCOD_%_removal'].values,

'PHA_yield': synthetic_df['PHA_yield'].values,

'mcl_PHA_%': synthetic_df['mcl_PHA_%'].values,

'scl_PHA_%': synthetic_df['scl_PHA_%'].values

}

synthetic_analysis_df = pd.DataFrame(synthetic_analysis)

\# Correlation matrix for synthetic test

print("\n1. CORRELATION MATRIX - SYNTHETIC SUBSTRATE TEST")

print("-"*100)

correlation_synthetic = synthetic_analysis_df.corr()

print(correlation_synthetic.to_string())

\# ============================================================================

\# STEP 3: SYNTHETIC SUBSTRATE TEST - REGRESSION ANALYSIS

\# ============================================================================

print("\n\n2. REGRESSION ANALYSES - SYNTHETIC SUBSTRATE TEST")

print("-"*100)

\# Regression 1: Ethanol % vs PHA yield

print("\nRegression 1: Ethanol Content (%) vs PHA Yield (g PHA/g VSS)")

x1 = synthetic_df['Ethanol_%'].values

y1 = synthetic_df['PHA_yield'].values

slope1, intercept1, r_value1, p_value1, std_err1 = linregress(x1, y1)

print(f" Equation: PHA_yield = {intercept1:.6f} + {slope1:.6f} × Ethanol_%")

print(f" R-value: {r_value1:.4f}")

print(f" R²: {r_value1**2:.4f}")

print(f" p-value: {p_value1:.4f}")

print(f" Significance: {'***' if p_value1 < 0.05 else 'ns'} (α = 0.05)")

print(f" Standard Error: {std_err1:.6f}")

\# Regression 2: Ethanol % vs Biomass increase

print("\nRegression 2: Ethanol Content (%) vs Biomass Increase (g VSS/L)")

x2 = synthetic_df['Ethanol_%'].values

y2 = synthetic_df['Biomass_increase'].values

slope2, intercept2, r_value2, p_value2, std_err2 = linregress(x2, y2)

print(f" Equation: Biomass_increase = {intercept2:.6f} + {slope2:.6f} × Ethanol_%")

print(f" R-value: {r_value2:.4f}")

print(f" R²: {r_value2**2:.4f}")

print(f" p-value: {p_value2:.4f}")

print(f" Significance: {'***' if p_value2 < 0.05 else 'ns'} (α = 0.05)")

print(f" Standard Error: {std_err2:.6f}")

\# Regression 3: Ethanol % vs sCOD removal

print("\nRegression 3: Ethanol Content (%) vs sCOD Removal (%)")

x3 = synthetic_df['Ethanol_%'].values

y3 = synthetic_df['sCOD_%_removal'].values

slope3, intercept3, r_value3, p_value3, std_err3 = linregress(x3, y3)

print(f" Equation: sCOD_removal = {intercept3:.6f} + {slope3:.6f} × Ethanol_%")

print(f" R-value: {r_value3:.4f}")

print(f" R²: {r_value3**2:.4f}")

print(f" p-value: {p_value3:.4f}")

print(f" Significance: {'***' if p_value3 < 0.05 else 'ns'} (α = 0.05)")

print(f" Standard Error: {std_err3:.6f}")

\# Regression 4: Ethanol % vs mcl-PHA %

print("\nRegression 4: Ethanol Content (%) vs mcl-PHA Composition (%)")

x4 = synthetic_df['Ethanol_%'].values

y4 = synthetic_df['mcl_PHA_%'].values

slope4, intercept4, r_value4, p_value4, std_err4 = linregress(x4, y4)

print(f" Equation: mcl_PHA_% = {intercept4:.6f} + {slope4:.6f} × Ethanol_%")

print(f" R-value: {r_value4:.4f}")

print(f" R²: {r_value4**2:.4f}")

print(f" p-value: {p_value4:.4f}")

print(f" Significance: {'***' if p_value4 < 0.05 else 'ns'} (α = 0.05)")

print(f" Standard Error: {std_err4:.6f}")

\# Summary table for synthetic substrate test

print("\n\n3. REGRESSION SUMMARY TABLE - SYNTHETIC SUBSTRATE TEST")

print("-"*100)

synthetic_regression_summary = pd.DataFrame({

'Variables': [

'Ethanol % → PHA Yield',

'Ethanol % → Biomass Increase',

'Ethanol % → sCOD Removal',

'Ethanol % → mcl-PHA %'

],

'Slope': [slope1, slope2, slope3, slope4],

'Intercept': [intercept1, intercept2, intercept3, intercept4],

'R-value': [r_value1, r_value2, r_value3, r_value4],

'R²': [r_value1**2, r_value2**2, r_value3**2, r_value4**2],

'p-value': [p_value1, p_value2, p_value3, p_value4],

'Std. Error': [std_err1, std_err2, std_err3, std_err4],

'Significance': [

'***' if p_value1 < 0.05 else 'ns',

'***' if p_value2 < 0.05 else 'ns',

'***' if p_value3 < 0.05 else 'ns',

'***' if p_value4 < 0.05 else 'ns'

]

})

print(synthetic_regression_summary.to_string(index=False))

\# ============================================================================

\# STEP 4: ACTUAL SUBSTRATE TEST - CORRELATION ANALYSIS

\# ============================================================================

print("\n\n" + "="*100)

print("ACTUAL SUBSTRATE TEST - CORRELATION AND REGRESSION ANALYSIS")

print("="*100)

\# Variables for analysis in actual test

actual_analysis = {

'F_M_ratio': actual_df['F_M_ratio'].values,

'Biomass_increase': actual_df['VSS_increase'].values,

'sCOD_removal_%': actual_df['COD_%_removal'].values,

'PHA_yield': actual_df['PHA_yield'].values,

'mcl_PHA_%': actual_df['mcl_PHA_%'].values,

'scl_PHA_%': actual_df['scl_PHA_%'].values

}

actual_analysis_df = pd.DataFrame(actual_analysis)

\# Correlation matrix for actual test

print("\n1. CORRELATION MATRIX - ACTUAL SUBSTRATE TEST")

print("-"*100)

correlation_actual = actual_analysis_df.corr()

print(correlation_actual.to_string())

\# ============================================================================

\# STEP 5: ACTUAL SUBSTRATE TEST - REGRESSION ANALYSIS

\# ============================================================================

print("\n\n2. REGRESSION ANALYSES - ACTUAL SUBSTRATE TEST")

print("-"*100)

\# Regression 1: F/M ratio vs PHA yield

print("\nRegression 1: F/M Ratio vs PHA Yield (g PHA/g VSS)")

x1_actual = actual_df['F_M_ratio'].values

y1_actual = actual_df['PHA_yield'].values

slope1_a, intercept1_a, r_value1_a, p_value1_a, std_err1_a = linregress(x1_actual, y1_actual)

print(f" Equation: PHA_yield = {intercept1_a:.6f} + {slope1_a:.6f} × F/M_ratio")

print(f" R-value: {r_value1_a:.4f}")

print(f" R²: {r_value1_a**2:.4f}")

print(f" p-value: {p_value1_a:.4f}")

print(f" Significance: {'***' if p_value1_a < 0.05 else 'ns'} (α = 0.05)")

print(f" Standard Error: {std_err1_a:.6f}")

\# Regression 2: F/M ratio vs Biomass increase

print("\nRegression 2: F/M Ratio vs Biomass Increase (g VSS/L)")

x2_actual = actual_df['F_M_ratio'].values

y2_actual = actual_df['VSS_increase'].values

slope2_a, intercept2_a, r_value2_a, p_value2_a, std_err2_a = linregress(x2_actual, y2_actual)

print(f" Equation: Biomass_increase = {intercept2_a:.6f} + {slope2_a:.6f} × F/M_ratio")

print(f" R-value: {r_value2_a:.4f}")

print(f" R²: {r_value2_a**2:.4f}")

print(f" p-value: {p_value2_a:.4f}")

print(f" Significance: {'***' if p_value2_a < 0.05 else 'ns'} (α = 0.05)")

print(f" Standard Error: {std_err2_a:.6f}")

\# Regression 3: F/M ratio vs sCOD removal

print("\nRegression 3: F/M Ratio vs sCOD Removal (%)")

x3_actual = actual_df['F_M_ratio'].values

y3_actual = actual_df['COD_%_removal'].values

slope3_a, intercept3_a, r_value3_a, p_value3_a, std_err3_a = linregress(x3_actual, y3_actual)

print(f" Equation: sCOD_removal = {intercept3_a:.6f} + {slope3_a:.6f} × F/M_ratio")

print(f" R-value: {r_value3_a:.4f}")

print(f" R²: {r_value3_a**2:.4f}")

print(f" p-value: {p_value3_a:.4f}")

print(f" Significance: {'***' if p_value3_a < 0.05 else 'ns'} (α = 0.05)")

print(f" Standard Error: {std_err3_a:.6f}")

\# Regression 4: F/M ratio vs mcl-PHA %

print("\nRegression 4: F/M Ratio vs mcl-PHA Composition (%)")

x4_actual = actual_df['F_M_ratio'].values

y4_actual = actual_df['mcl_PHA_%'].values

slope4_a, intercept4_a, r_value4_a, p_value4_a, std_err4_a = linregress(x4_actual, y4_actual)

print(f" Equation: mcl_PHA_% = {intercept4_a:.6f} + {slope4_a:.6f} × F/M_ratio")

print(f" R-value: {r_value4_a:.4f}")

print(f" R²: {r_value4_a**2:.4f}")

print(f" p-value: {p_value4_a:.4f}")

print(f" Significance: {'***' if p_value4_a < 0.05 else 'ns'} (α = 0.05)")

print(f" Standard Error: {std_err4_a:.6f}")

\# Regression 5: F/M ratio vs scl-PHA %

print("\nRegression 5: F/M Ratio vs scl-PHA Composition (%)")

x5_actual = actual_df['F_M_ratio'].values

y5_actual = actual_df['scl_PHA_%'].values

slope5_a, intercept5_a, r_value5_a, p_value5_a, std_err5_a = linregress(x5_actual, y5_actual)

print(f" Equation: scl_PHA_% = {intercept5_a:.6f} + {slope5_a:.6f} × F/M_ratio")

print(f" R-value: {r_value5_a:.4f}")

print(f" R²: {r_value5_a**2:.4f}")

print(f" p-value: {p_value5_a:.4f}")

print(f" Significance: {'***' if p_value5_a < 0.05 else 'ns'} (α = 0.05)")

print(f" Standard Error: {std_err5_a:.6f}")

\# Summary table for actual substrate test

print("\n\n3. REGRESSION SUMMARY TABLE - ACTUAL SUBSTRATE TEST")

print("-"*100)

actual_regression_summary = pd.DataFrame({

'Variables': [

'F/M Ratio → PHA Yield',

'F/M Ratio → Biomass Increase',

'F/M Ratio → sCOD Removal',

'F/M Ratio → mcl-PHA %',

'F/M Ratio → scl-PHA %'

],

'Slope': [slope1_a, slope2_a, slope3_a, slope4_a, slope5_a],

'Intercept': [intercept1_a, intercept2_a, intercept3_a, intercept4_a, intercept5_a],

'R-value': [r_value1_a, r_value2_a, r_value3_a, r_value4_a, r_value5_a],

'R²': [r_value1_a**2, r_value2_a**2, r_value3_a**2, r_value4_a**2, r_value5_a**2],

'p-value': [p_value1_a, p_value2_a, p_value3_a, p_value4_a, p_value5_a],

'Std. Error': [std_err1_a, std_err2_a, std_err3_a, std_err4_a, std_err5_a],

'Significance': [

'***' if p_value1_a < 0.05 else 'ns',

'***' if p_value2_a < 0.05 else 'ns',

'***' if p_value3_a < 0.05 else 'ns',

'***' if p_value4_a < 0.05 else 'ns',

'***' if p_value5_a < 0.05 else 'ns'

]

})

print(actual_regression_summary.to_string(index=False))

\# ============================================================================

\# STEP 6: VISUALIZATION - REGRESSION PLOTS

\# ============================================================================

print("\n\n" + "="*100)

print("GENERATING VISUALIZATION PLOTS")

print("="*100)

\# Set style

sns.set_style("whitegrid")

\# Synthetic Substrate Test - Regression Plots

fig1, axes = plt.subplots(2, 2, figsize=(14, 10))

fig1.suptitle('Synthetic Substrate Test - Regression Analysis', fontsize=16, fontweight='bold')

color_syn = '#1f77b4'

\# Plot 1: Ethanol % vs PHA Yield

ax = axes[0, 0]

ax.scatter(x1, y1, s=100, color=color_syn, alpha=0.7, edgecolors='black', linewidth=1.5)

x_line = np.array([0, 100])

y_line = intercept1 + slope1 * x_line

ax.plot(x_line, y_line, 'r--', linewidth=2, label=f'y = {intercept1:.4f} + {slope1:.4f}x')

ax.set_xlabel('Ethanol Content (%)', fontweight='bold')

ax.set_ylabel('PHA Yield (g PHA/g VSS)', fontweight='bold')

ax.set_title(f'Ethanol % → PHA Yield (R² = {r_value1**2:.4f}, p = {p_value1:.4f})', fontweight='bold')

ax.legend(fontsize=9)

ax.grid(True, alpha=0.3)

\# Plot 2: Ethanol % vs Biomass Increase

ax = axes[0, 1]

ax.scatter(x2, y2, s=100, color=color_syn, alpha=0.7, edgecolors='black', linewidth=1.5)

x_line = np.array([0, 100])

y_line = intercept2 + slope2 * x_line

ax.plot(x_line, y_line, 'r--', linewidth=2, label=f'y = {intercept2:.4f} + {slope2:.4f}x')

ax.set_xlabel('Ethanol Content (%)', fontweight='bold')

ax.set_ylabel('Biomass Increase (g VSS/L)', fontweight='bold')

ax.set_title(f'Ethanol % → Biomass Increase (R² = {r_value2**2:.4f}, p = {p_value2:.4f})', fontweight='bold')

ax.legend(fontsize=9)

ax.grid(True, alpha=0.3)

\# Plot 3: Ethanol % vs sCOD Removal

ax = axes[1, 0]

ax.scatter(x3, y3, s=100, color=color_syn, alpha=0.7, edgecolors='black', linewidth=1.5)

x_line = np.array([0, 100])

y_line = intercept3 + slope3 * x_line

ax.plot(x_line, y_line, 'r--', linewidth=2, label=f'y = {intercept3:.4f} + {slope3:.4f}x')

ax.set_xlabel('Ethanol Content (%)', fontweight='bold')

ax.set_ylabel('sCOD Removal (%)', fontweight='bold')

ax.set_title(f'Ethanol % → sCOD Removal (R² = {r_value3**2:.4f}, p = {p_value3:.4f})', fontweight='bold')

ax.legend(fontsize=9)

ax.grid(True, alpha=0.3)

\# Plot 4: Ethanol % vs mcl-PHA %

ax = axes[1, 1]

ax.scatter(x4, y4, s=100, color=color_syn, alpha=0.7, edgecolors='black', linewidth=1.5)

x_line = np.array([0, 100])

y_line = intercept4 + slope4 * x_line

ax.plot(x_line, y_line, 'r--', linewidth=2, label=f'y = {intercept4:.4f} + {slope4:.4f}x')

ax.set_xlabel('Ethanol Content (%)', fontweight='bold')

ax.set_ylabel('mcl-PHA Composition (%)', fontweight='bold')

ax.set_title(f'Ethanol % → mcl-PHA % (R² = {r_value4**2:.4f}, p = {p_value4:.4f})', fontweight='bold')

ax.legend(fontsize=9)

ax.grid(True, alpha=0.3)

plt.tight_layout()

plt.savefig('synthetic_regression_analysis.png', dpi=300, bbox_inches='tight')

print("✓ Synthetic substrate regression plot saved")

plt.close()

\# Actual Substrate Test - Regression Plots

fig2, axes = plt.subplots(2, 3, figsize=(16, 10))

fig2.suptitle('Actual Substrate Test (P-FWW) - Regression Analysis', fontsize=16, fontweight='bold')

color_act = '#ff7f0e'

\# Plot 1: F/M Ratio vs PHA Yield

ax = axes[0, 0]

ax.scatter(x1_actual, y1_actual, s=100, color=color_act, alpha=0.7, edgecolors='black', linewidth=1.5)

x_line = np.linspace(x1_actual.min() - 5, x1_actual.max() + 5, 100)

y_line = intercept1_a + slope1_a * x_line

ax.plot(x_line, y_line, 'r--', linewidth=2, label=f'y = {intercept1_a:.4f} + {slope1_a:.4f}x')

ax.set_xlabel('F/M Ratio (g COD/g VSS·day)', fontweight='bold')

ax.set_ylabel('PHA Yield (g PHA/g VSS)', fontweight='bold')

ax.set_title(f'F/M → PHA Yield (R² = {r_value1_a**2:.4f}, p = {p_value1_a:.4f})', fontweight='bold')

ax.legend(fontsize=9)

ax.grid(True, alpha=0.3)

\# Plot 2: F/M Ratio vs Biomass Increase

ax = axes[0, 1]

ax.scatter(x2_actual, y2_actual, s=100, color=color_act, alpha=0.7, edgecolors='black', linewidth=1.5)

x_line = np.linspace(x2_actual.min() - 5, x2_actual.max() + 5, 100)

y_line = intercept2_a + slope2_a * x_line

ax.plot(x_line, y_line, 'r--', linewidth=2, label=f'y = {intercept2_a:.4f} + {slope2_a:.4f}x')

ax.set_xlabel('F/M Ratio (g COD/g VSS·day)', fontweight='bold')

ax.set_ylabel('Biomass Increase (g VSS/L)', fontweight='bold')

ax.set_title(f'F/M → Biomass Increase (R² = {r_value2_a**2:.4f}, p = {p_value2_a:.4f})***', fontweight='bold', color='darkgreen')

ax.legend(fontsize=9)

ax.grid(True, alpha=0.3)

\# Plot 3: F/M Ratio vs sCOD Removal

ax = axes[0, 2]

ax.scatter(x3_actual, y3_actual, s=100, color=color_act, alpha=0.7, edgecolors='black', linewidth=1.5)

x_line = np.linspace(x3_actual.min() - 5, x3_actual.max() + 5, 100)

y_line = intercept3_a + slope3_a * x_line

ax.plot(x_line, y_line, 'r--', linewidth=2, label=f'y = {intercept3_a:.4f} + {slope3_a:.4f}x')

ax.set_xlabel('F/M Ratio (g COD/g VSS·day)', fontweight='bold')

ax.set_ylabel('sCOD Removal (%)', fontweight='bold')

ax.set_title(f'F/M → sCOD Removal (R² = {r_value3_a**2:.4f}, p = {p_value3_a:.4f})', fontweight='bold')

ax.legend(fontsize=9)

ax.grid(True, alpha=0.3)

\# Plot 4: F/M Ratio vs mcl-PHA %

ax = axes[1, 0]

ax.scatter(x4_actual, y4_actual, s=100, color=color_act, alpha=0.7, edgecolors='black', linewidth=1.5)

x_line = np.linspace(x4_actual.min() - 5, x4_actual.max() + 5, 100)

y_line = intercept4_a + slope4_a * x_line

ax.plot(x_line, y_line, 'r--', linewidth=2, label=f'y = {intercept4_a:.4f} + {slope4_a:.4f}x')

ax.set_xlabel('F/M Ratio (g COD/g VSS·day)', fontweight='bold')

ax.set_ylabel('mcl-PHA Composition (%)', fontweight='bold')

ax.set_title(f'F/M → mcl-PHA % (R² = {r_value4_a**2:.4f}, p = {p_value4_a:.4f})***', fontweight='bold', color='darkgreen')

ax.legend(fontsize=9)

ax.grid(True, alpha=0.3)

\# Plot 5: F/M Ratio vs scl-PHA %

ax = axes[1, 1]

ax.scatter(x5_actual, y5_actual, s=100, color=color_act, alpha=0.7, edgecolors='black', linewidth=1.5)

x_line = np.linspace(x5_actual.min() - 5, x5_actual.max() + 5, 100)

y_line = intercept5_a + slope5_a * x_line

ax.plot(x_line, y_line, 'r--', linewidth=2, label=f'y = {intercept5_a:.4f} + {slope5_a:.4f}x')

ax.set_xlabel('F/M Ratio (g COD/g VSS·day)', fontweight='bold')

ax.set_ylabel('scl-PHA Composition (%)', fontweight='bold')

ax.set_title(f'F/M → scl-PHA % (R² = {r_value5_a**2:.4f}, p = {p_value5_a:.4f})***', fontweight='bold', color='darkgreen')

ax.legend(fontsize=9)

ax.grid(True, alpha=0.3)

fig2.delaxes(axes[1, 2])

plt.tight_layout()

plt.savefig('actual_regression_analysis.png', dpi=300, bbox_inches='tight')

print("✓ Actual substrate regression plot saved")

plt.close()

\# Correlation Heatmaps

fig3, axes = plt.subplots(1, 2, figsize=(14, 5))

fig3.suptitle('Correlation Matrices - Synthetic vs Actual Substrate Tests', fontsize=14, fontweight='bold')

sns.heatmap(correlation_synthetic, annot=True, fmt='.3f', cmap='coolwarm', center=0,

cbar_kws={'label': 'Correlation Coefficient'}, ax=axes[0], vmin=-1, vmax=1,

square=True, linewidths=0.5)

axes[0].set_title('Synthetic Substrate Test', fontweight='bold', fontsize=12)

sns.heatmap(correlation_actual, annot=True, fmt='.3f', cmap='coolwarm', center=0,

cbar_kws={'label': 'Correlation Coefficient'}, ax=axes[1], vmin=-1, vmax=1,

square=True, linewidths=0.5)

axes[1].set_title('Actual Substrate Test (P-FWW)', fontweight='bold', fontsize=12)

plt.tight_layout()

plt.savefig('correlation_heatmaps.png', dpi=300, bbox_inches='tight')

print("✓ Correlation heatmaps saved")

plt.close()

print("\n✓ All visualization plots generated successfully!")

\# ============================================================================

\# STEP 7: EXPORT RESULTS TO CSV

\# ============================================================================

print("\n" + "="*100)

print("EXPORTING RESULTS TO CSV FILES")

print("="*100)

synthetic_regression_summary.to_csv('synthetic_regression_summary.csv', index=False)

print("✓ synthetic_regression_summary.csv")

actual_regression_summary.to_csv('actual_regression_summary.csv', index=False)

print("✓ actual_regression_summary.csv")

correlation_synthetic.to_csv('synthetic_correlation_matrix.csv')

print("✓ synthetic_correlation_matrix.csv")

correlation_actual.to_csv('actual_correlation_matrix.csv')

print("✓ actual_correlation_matrix.csv")

print("\n✓ ALL ANALYSIS COMPLETE!")

print("="*100)

## S1.2 Analysis reports

After executing the code, the following report was generated in the kernel:

Data loaded successfully!

Synthetic test shape: (3, 14)

Actual test shape: (6, 10)

====================================================================================================

SYNTHETIC SUBSTRATE TEST - CORRELATION AND REGRESSION ANALYSIS

====================================================================================================

1. CORRELATION MATRIX - SYNTHETIC SUBSTRATE TEST

----------------------------------------------------------------------------------------------------

Ethanol_% Biomass_increase sCOD_removal PHA_yield mcl_PHA_% scl_PHA_%

Ethanol_%	1.000000	0.211914	-0.420583	0.050900	0.902273 -0.902273

Biomass_increase	0.211914	1.000000	0.797521	0.986808 -0.230169	0.230169

sCOD_removal	-0.420583	0.797521	1.000000	0.884670 -0.770658	0.770658

PHA_yield	0.050900	0.986808	0.884670	1.000000 -0.384681	0.384681

mcl_PHA_%	0.902273	-0.230169	-0.770658 -0.384681	1.000000 -1.000000

scl_PHA_%	-0.902273	0.230169	0.770658	0.384681 -1.000000	1.000000

2. REGRESSION ANALYSES - SYNTHETIC SUBSTRATE TEST

----------------------------------------------------------------------------------------------------

Regression 1: Ethanol Content (%) vs PHA Yield (g PHA/g VSS)

Equation: PHA_yield = 0.535073 + 0.000136 × Ethanol_%

R-value: 0.0509

R²: 0.0026

p-value: 0.9676

Significance: ns (α = 0.05)

Standard Error: 0.002673

Regression 2: Ethanol Content (%) vs Biomass Increase (g VSS/L)

Equation: Biomass_increase = 0.954923 + 0.003152 × Ethanol_%

R-value: 0.2119

R²: 0.0449

p-value: 0.8641

Significance: ns (α = 0.05)

Standard Error: 0.014535

Regression 3: Ethanol Content (%) vs sCOD Removal (%)

Equation: sCOD_removal = 31.515105 + -0.114921 × Ethanol_%

R-value: -0.4206

R²: 0.1769

p-value: 0.7237

Significance: ns (α = 0.05)

Standard Error: 0.247901

Regression 4: Ethanol Content (%) vs mcl-PHA Composition (%)

Equation: mcl_PHA_% = 91.121649 + 0.073061 × Ethanol_%

R-value: 0.9023

R²: 0.8141

p-value: 0.2838

Significance: ns (α = 0.05)

Standard Error: 0.034913

3. REGRESSION SUMMARY TABLE - SYNTHETIC SUBSTRATE TEST

----------------------------------------------------------------------------------------------------

Variables	Slope Intercept	R-value	R² p-value Std. Error Significance

Ethanol % → PHA Yield 0.000136	0.535073 0.050900 0.002591 0.967582	0.002673	ns

Ethanol % → Biomass Increase 0.003152	0.954923 0.211914 0.044907 0.864061	0.014535	ns

Ethanol % → sCOD Removal -0.114921 31.515105 -0.420583 0.176890 0.723651	0.247901	ns

Ethanol % → mcl-PHA % 0.073061 91.121649 0.902273 0.814096 0.283795	0.034913	ns

====================================================================================================

ACTUAL SUBSTRATE TEST - CORRELATION AND REGRESSION ANALYSIS

====================================================================================================

1. CORRELATION MATRIX - ACTUAL SUBSTRATE TEST

----------------------------------------------------------------------------------------------------

F_M_ratio Biomass_increase sCOD_removal_% PHA_yield mcl_PHA_% scl_PHA_%

F_M_ratio	1.000000	0.987582	-0.714717	0.247006	0.903040 -0.903040

Biomass_increase	0.987582	1.000000	-0.708996	0.220378	0.890303 -0.890303

sCOD_removal_%	-0.714717	-0.708996	1.000000 -0.679593 -0.875032	0.875032

PHA_yield	0.247006	0.220378	-0.679593	1.000000	0.291142 -0.291142

mcl_PHA_%	0.903040	0.890303	-0.875032	0.291142	1.000000 -1.000000

scl_PHA_%	-0.903040	-0.890303	0.875032 -0.291142 -1.000000	1.000000

2. REGRESSION ANALYSES - ACTUAL SUBSTRATE TEST

----------------------------------------------------------------------------------------------------

Regression 1: F/M Ratio vs PHA Yield (g PHA/g VSS)

Equation: PHA_yield = 0.232036 + 0.000407 × F/M_ratio

R-value: 0.2470

R²: 0.0610

p-value: 0.6370

Significance: ns (α = 0.05)

Standard Error: 0.000798

Regression 2: F/M Ratio vs Biomass Increase (g VSS/L)

Equation: Biomass_increase = 3.466686 + 0.132830 × F/M_ratio

R-value: 0.9876

R²: 0.9753

p-value: 0.0002

Significance: *** (α = 0.05)

Standard Error: 0.010565

Regression 3: F/M Ratio vs sCOD Removal (%)

Equation: sCOD_removal = 79.766845 + -0.164961 × F/M_ratio

R-value: -0.7147

R²: 0.5108

p-value: 0.1105

Significance: ns (α = 0.05)

Standard Error: 0.080715

Regression 4: F/M Ratio vs mcl-PHA Composition (%)

Equation: mcl_PHA_% = 17.366940 + 0.568496 × F/M_ratio

R-value: 0.9030

R²: 0.8155

p-value: 0.0136

Significance: *** (α = 0.05)

Standard Error: 0.135211

Regression 5: F/M Ratio vs scl-PHA Composition (%)

Equation: scl_PHA_% = 82.633060 + -0.568496 × F/M_ratio

R-value: -0.9030

R²: 0.8155

p-value: 0.0136

Significance: *** (α = 0.05)

Standard Error: 0.135211

3. REGRESSION SUMMARY TABLE - ACTUAL SUBSTRATE TEST

----------------------------------------------------------------------------------------------------

Variables	Slope Intercept	R-value	R² p-value Std. Error Significance

F/M Ratio → PHA Yield 0.000407	0.232036 0.247006 0.061012 0.637026	0.000798	ns

F/M Ratio → Biomass Increase 0.132830	3.466686 0.987582 0.975317 0.000230	0.010565	***

F/M Ratio → sCOD Removal -0.164961 79.766845 -0.714717 0.510821 0.110470	0.080715	ns

F/M Ratio → mcl-PHA % 0.568496 17.366940 0.903040 0.815481 0.013646	0.135211	***

F/M Ratio → scl-PHA % -0.568496 82.633060 -0.903040 0.815481 0.013646	0.135211	***

====================================================================================================

GENERATING VISUALIZATION PLOTS

====================================================================================================

✓ Synthetic substrate regression plot saved

✓ Actual substrate regression plot saved

✓ Correlation heatmaps saved

✓ All visualization plots generated successfully!

====================================================================================================

EXPORTING RESULTS TO CSV FILES

====================================================================================================

✓ synthetic_regression_summary.csv

✓ actual_regression_summary.csv

✓ synthetic_correlation_matrix.csv

✓ actual_correlation_matrix.csv

✓ ALL ANALYSIS COMPLETE!

Additionally, after executing the codebase, several .csv files and visualization plots can be generated based according to the description in the analysis report.

**Table S1**. Generated data from *synthetic_regression_summary.csv*

|  | Variables | Slope | Intercept | R-value | R² | p-value | Std. Error | Significance |
| --- | --- | --- | --- | --- | --- | --- | --- | --- |
| 1 | Ethanol % → PHA Yield | 0.000136 | 0.535073 | 0.0509 | 0.002591 | 0.967582 | 0.002673 | ns |
| 2 | Ethanol % → Biomass Increase | 0.003152 | 0.954923 | 0.211914 | 0.044907 | 0.864061 | 0.014535 | ns |
| 3 | Ethanol % → sCOD Removal | -0.11492 | 31.5151 | -0.42058 | 0.17689 | 0.723651 | 0.247901 | ns |
| 4 | Ethanol % → mcl-PHA % | 0.073061 | 91.12165 | 0.902273 | 0.814096 | 0.283795 | 0.034913 | ns |

**Table S2**. Generated data from *actual_regression_summary.csv*

|  | Variables | Slope | Intercept | R-value | R² | p-value | Std. Error | Significance |
| --- | --- | --- | --- | --- | --- | --- | --- | --- |
| 1 | F/M Ratio → PHA Yield | 0.000407 | 0.232036 | 0.247006 | 0.061012 | 0.637026 | 0.000798 | ns |
| 2 | F/M Ratio → Biomass Increase | 0.13283 | 3.466686 | 0.987582 | 0.975317 | 0.00023 | 0.010565 | *** |
| 3 | F/M Ratio → sCOD Removal | -0.16496 | 79.76684 | -0.71472 | 0.510821 | 0.11047 | 0.080715 | ns |
| 4 | F/M Ratio → mcl-PHA % | 0.568496 | 17.36694 | 0.90304 | 0.815481 | 0.013646 | 0.135211 | *** |
| 5 | F/M Ratio → scl-PHA % | -0.5685 | 82.63306 | -0.90304 | 0.815481 | 0.013646 | 0.135211 | *** |

**Table S3**. Generated data from *synthetic_correlation_matrix.csv*

|  |  | Ethanol_% | Biomass_increase | sCOD_removal | PHA_yield | mcl_PHA_% | scl_PHA_% |
| --- | --- | --- | --- | --- | --- | --- | --- |
| 1 | Ethanol_% | 1 | 0.211914 | -0.42058 | 0.0509 | 0.902273 | -0.90227 |
| 2 | Biomass_increase | 0.211914 | 1 | 0.797521 | 0.986808 | -0.23017 | 0.230169 |
| 3 | sCOD_removal | -0.42058 | 0.797521 | 1 | 0.88467 | -0.77066 | 0.770658 |
| 4 | PHA_yield | 0.0509 | 0.986808 | 0.88467 | 1 | -0.38468 | 0.384681 |
| 5 | mcl_PHA_% | 0.902273 | -0.23017 | -0.77066 | -0.38468 | 1 | -1 |
| 6 | scl_PHA_% | -0.90227 | 0.230169 | 0.770658 | 0.384681 | -1 | 1 |

**Table S4**. Generated data from *actual_correlation_matrix.csv*

|  |  | F_M_ratio | Biomass_increase | sCOD_removal_% | PHA_yield | mcl_PHA_% | scl_PHA_% |
| --- | --- | --- | --- | --- | --- | --- | --- |
| 1 | F_M_ratio | 1 | 0.987582 | -0.71472 | 0.247006 | 0.90304 | -0.90304 |
| 2 | Biomass_increase | 0.987582 | 1 | -0.709 | 0.220378 | 0.890303 | -0.8903 |
| 3 | sCOD_removal_% | -0.71472 | -0.709 | 1 | -0.67959 | -0.87503 | 0.875032 |
| 4 | PHA_yield | 0.247006 | 0.220378 | -0.67959 | 1 | 0.291142 | -0.29114 |
| 5 | mcl_PHA_% | 0.90304 | 0.890303 | -0.87503 | 0.291142 | 1 | -1 |
| 6 | scl_PHA_% | -0.90304 | -0.8903 | 0.875032 | -0.29114 | -1 | 1 |

**Figure S1**. Regression plots derived from the synthetic substrate test.

**Figure S2**. Regression plot derived from the actual substrate test.
